# Sex differences in prognostic role of fasting glucose, Oral glucose tolerance, and HbA1c in diabetic cardiovascular disease

**DOI:** 10.1111/1753-0407.13358

**Published:** 2023-02-14

**Authors:** Yilin Yoshida, Zhipeng Chen, Robin L. Baudier, Marie Krousel‐Wood, Amanda H. Anderson, Vivian A. Fonseca, Franck Mauvais‐Jarvis

**Affiliations:** ^1^ Section of Endocrinology and Metabolism, Deming Department of Medicine Tulane University School of Medicine New Orleans Louisiana USA; ^2^ Tulane Center of Excellence in Sex‐Based Biology & Medicine Tulane University School of Medicine New Orleans Louisiana USA; ^3^ Southeast Louisiana VA Medical Center New Orleans Louisiana USA; ^4^ Department of Biostatistics and Data Science Tulane University School of Public Health and Tropical Medicine New Orleans Louisiana USA; ^5^ Department of Epidemiology Tulane University School of Public Health and Tropical Medicine New Orleans Louisiana USA; ^6^ Section of General Internal Medicine, Deming Department of Medicine Tulane University School of Medicine New Orleans Louisiana USA

**Keywords:** diabetic cardiovascular complications, fasting glucose, HbA1c, oral glucose tolerance, sex difference

## Abstract

**Highlights**

- Fasting glucose‐defined prediabetes and undiagnosed diabetes based on the American Diabetes Association criteria were associated with a greater risk of coronary heart disease, stroke, and composite atherosclerotic cardiovascular disease in women. In contrast, oral glucose tolerance‐defined prediabetes and undiagnosed diabetes were associated with a greater risk of all cardiovascular outcomes in men.
- Intermediate A1c was associated with a more pronounced effect on the risk of coronary heart disease and stroke in women, whereas the above diagnostic level of A1c was associated with a higher magnitude of coronary heart risk in undiagnosed men but a higher magnitude of stroke risk in undiagnosed women.

To the Editor

The prognostic role of fasting glucose (FG), glycated hemoglobin A1c (A1c), and oral glucose tolerance (OGT) in diabetic cardiovascular disease (CVD) remains controversial. Some studies support that OGT is a better predictor of macrovascular complications.^1^, ^2^ Other evidence suggests that compared to impaired glucose tolerance (IGT), A1c and impaired fasting glucose (IFG) better discriminate the risk for diabetic cardiovascular complications and mortality.^3^, ^4^ Notably, the sex differences in CVD risk associated with elevated FG, A1c, and OGT are rarely reported. Men and women differ in glucose homeostasis.^5^ Men have a higher prevalence of IFG. In contrast, women have a higher prevalence of IGT, which is likely attributable to women's delayed glucose absorption compared with men's.^6^

There was also a sex disparity in diabetic CVD.^7^ Compared to men with diabetes, female patients have a 58% and an 8% higher mortality risk for coronary heart disease and stroke, respectively.^8^ However, these risk estimates were primarily based on diagnosed diabetes. The extent to which prediabetes and undiagnosed diabetes were associated with the risk of CVD in men and women remains unclear. It is also unclear whether the associations varied across glycemic measures. In this study, we sought to assess sex differences between prediabetes and undiagnosed diabetes measured by isolated FG, A1c, and OGT and the risk of CVD.

## METHODS

We performed a prospective cohort analysis of men and women without diabetes diagnoses or prevalent CVD from the Atherosclerosis Risk in Communities (ARIC) Study visits 2–6 (1990–2017) in the United States.^9^ We identified individuals with pre‐diabetes with FG 100–125 mg/dL (IFG), A1c 5.7%–6.4%, or OGT 140–199 mg/dL (IGT), and individuals with undiagnosed diabetes with FG ≥126 mg/dL, OGT ≥200 mg/dL, or A1c ≥6.5% based on the American Diabetes Association (ADA) criteria.^10^ CVD outcomes included incident coronary heart disease (CHD) comprising myocardial infarction, CHD death, cardiac procedures, ischemic or hemorrhagic stroke, and a composite atherosclerotic cardiovascular disease (ASCVD), including CHD or stroke.

We performed Cox proportional hazards models to evaluate the association of FG, A1c, and OGT in the prediabetic and diabetic ranges and the risk of CVD. We adjusted for age, race/ethnicity, education, body mass index, smoking, blood pressure, cholesterols, menopausal status (women only), uric acids, and antihypertensive or lipid‐lowering medications. We tested the sex modifying effect of the association by adding interaction terms with *p* ≤ .1 considered significant.

## RESULTS

**FIGURE 1:**
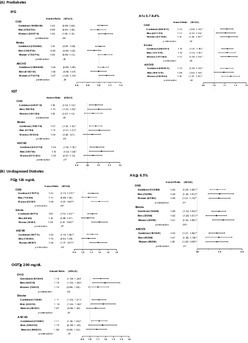
Risk of cardiovascular disease associated with glycemic measures (A). Risk of cardiovascular disease associated with impaired fasting glucose, HbA1c 5.7%–6.4%, and impaired glucose tolerance (B). Risk of cardiovascular disease associated with fasting glucose≥126 mg/dL, HbA1c ≥ 6.5%, and oral glucose tolerance ≥200 mg/dL in undiagnosed individuals. * < .05; ** < .0001. Adjusted for age, race/ethnicity, education, body mass index, smoking, blood pressure, cholesterols, menopausal status, uric acids, and antihypertensive or lipid‐lowering medications. ASCVD, atherosclerotic cardiovascular disease; CHD, coronary heart disease; CI, confidence interval; FG, fasting glucose; HbA1C, glycated hemoglobin; IFG, impaired fasting glucose; IGT, impaired glucose tolerance; OGT, oral glucose tolerance.

A total of 11 262 individuals were included, of whom 6409 (57%) were women and 8800 (78%) were non‐Hispanic White (Table 1). In the median follow‐up of 23 years, IFG was associated with a significant risk of CHD, stroke, and ASCVD in women but not men. The association between IFG and stroke showed a sex difference (Figure 1A). Prediabetic level of A1c was associated with a significant risk of all CVD outcomes in both sexes, with a more pronounced effect on ASCVD in women (Figure 1A). IGT was associated with an increased risk of all CVD outcomes in both sexes, with men showing a higher risk than women (Figure 1A). Above ADA diagnostic threshold of FG was associated with a higher magnitude of CVD risk in undiagnosed women than in men (Figure 1B), whereas elevated OGT was associated with greater CVD risk among undiagnosed men (Figure 1B). Above diagnostic level of A1c was associated higher magnitude of CHD risk in undiagnosed men but a higher magnitude of stroke risk in undiagnosed women. Racial stratified results are generally consistent with the main results (Figure S1).

**TABLE 1:** Baseline characteristics

	All *N* = 11 262	Men *N* = 4853	Women *N* = 6409	*p* value
**Age (mean ± SD, years)**	56.8 ± 5.71	57.1 ± 5.72	56.5 ± 5.68	<.0001
**Race/ethnicity (*n*, %)**				<.0001
Non‐Hispanic White	8800 (78.14%)	3932 (81.02%)	4868 (75.96%)	
Non‐Hispanic Black	2462 (21.86%)	921 (18.98%)	1541 (24.04%)	
**Low education** (< high school) (*n*, %)	865 (7.68%)	460 (9.48%)	405 (6.32%)	<.0001
**Obesity** (BMI ≥30) (*n*, %, kg/m^2^)	2978 (26.44%)	1131 (23.31%)	1847 (28.82%)	<.0001
**High cholesterol** (total chol ≥200 mg/dL) (*n*, %)	6502 (57.73%)	2530 (52.13%)	3972 (61.98%)	<.0001
**Hypertension** (systolic ≥140; diastolic ≥90 mm Hg (*n*, %)	1760 (15.63%)	833 (17.16%)	927 (14.46%)	<.0001
**Current smoker** (*n*, %)	2564 (22.77%)	1195 (24.62%)	1369 (21.36%)	<.0001
**Antihypertensive medications** (*n*, %)	3214 (28.54%)	1279 (26.35%)	1935 (30.19%)	<.0001
**Lipid‐lowering medications** (*n*, %)	230 (2.04%)	92 (1.90%)	138 (2.15%)	0.3442
**Menopausal status** ^a^				
Premenopause (*n*, %)	NA	NA	1302 (20.32%)	NA
Perimenopause (*n*, %)	NA	NA	601 (9.38%)	NA
Postmenopause (*n*, %)	NA	NA	3700 (57.73%)	NA
**Uric acid (mean, mg/dL)**	6.4 ± 1.6	5.8 ± 1.42	7.2 ± 1.49	<.0001
**Uric acid >7.2 mg/dL (*n*, %)**	3113 (27.64%)	943 (14.71%)	2170 (44.71%)	<.0001
**Prediabetes**				
HbA1c 5.7–6.4% (*n*, %)	3013 (26.75%)	1370 (28.23%)	1643 (25.64%)	0.0028
Fasting glucose 100–125 mg/dL (*n*, %)	5450 (48.39%)	2734 (56.34%)	2716 (42.38%)	<.0001
Oral glucose tolerance 140–199 mg/dL (*n*, %)	2118 (18.81%)	794 (16.36%)	1324 (20.66%)	<.0001
**Undiagnosed diabetes**				
HbA1c ≥ 6.5% (*n*, %)	500 (4.44%)	206 (4.24%)	294 (4.59%)	.6327
Fasting glucose ≥126 mg/dL (*n*, %)	713 (6.33%)	346 (7.13%)	367 (5.73%)	<.0001
Oral glucose tolderance ≥200 mg/dL (*n*, %)	841 (7.47%)	310 (6.39%)	531 (8.29%)	<.0001

^a^ Menopausal status was defined by the following questions. Women were asked, “Have you had any menstrual periods during the past 2 years?” and “Have you reached menopause?” They were also asked questions related to the surgical removal of ovaries and uterus. Women who had a menstrual period within the previous 2 years but denied current menopause were classified as “premenopausal.” Women who had a menstrual period within the previous 2 years and answered “yes” or “uncertain” to the question regarding current menopause were classified as “perimenopausal.” Women who had not had a menstrual period within the previous 2 years and did not have surgical removal of ovaries or uterus were classified as natural “postmenopausal.”

Abbreviations: BMI, body mass index; HbA1C, glycated hemoglobin.

## COMMENT

Our study extended previous findings about glycemic measures and risk of CVD by presenting risk stratification by sex using a biracial cohort with 2 decades of follow‐up. We found that A1c‐ and FG‐defined prediabetes were associated with a greater risk of all CVD outcomes in women. In contrast, IGT was associated with a stronger CVD risk in men. Above diagnostic levels of FG and OGT also showed same pattern of sex differences about CVD risk. An earlier analysis based on the Diabetes Epidemiology: Collaborative Analysis of Diagnostic Criteria in Europe (DECODE) study also indicated that OGT was a better predictor of stroke than FG in men, whereas FG was a better predictor in women.^11^ Additionally, the Rancho Bernardo Study in California found that the A1c‐defined prediabetes but not IGT was associated with CVD mortality in women only.^4^

There are pathophysiological differences between FG, OGT, and A1c. IFG expresses increased hepatic glucose production and impaired insulin secretion, whereas IGT expresses peripheral insulin resistance and impairment of insulin responses. A1c encompasses glycemic control in the fasting and postprandial states, reflecting chronic hyperglycemia.^11^ However, the pathophysiological differences in the glycemic measures related to the sex differences in diabetic CVD are unclear. But it may be related to sex differences in pancreatic endocrine function (eg, women have a greater insulin secretion capacity in the postprandial state)^6^ and sex steroid hormones and hormone‐associated cardiometabolic effects.^6^

Our study has several limitations. OGT was measured at a later time point (median follow‐up 15 years for OGT vs 23 years for FG and A1c). Additionally, owing to data unavailability, we cannot assess the effect of undiagnosed period. Also, it is possible that some prediabetes patients progressed into diabetes in the long follow‐up period; thus CVD risk may be a mixed effect of prediabetes and diabetes.

From this large longitudinal cohort in the United States, we observed sex differences in glycemic measures and CVD risk among individuals without diabetes diagnoses. FG and A1c may play a more prominent prognostic role in the risk of CHD and stroke in women, whereas OGT may better predict stroke risk in men. These differences should be considered in routine testing to better assess future risk of CVD.

## AUTHOR CONTRIBUTIONS

Yoshida is the guarantor of this work and, as such, had full access to all the data in the study and takes responsibility for the integrity of the data and the accuracy of the data analysis. Yoshida designed the study and contributed to the data management and analysis, the result interpretation, and the drafting, reviewing, and editing the manuscript. Chen contributed to data management, analysis, and figure and table making. Baudier provided statistical advice and contributed to manuscript reviewing and editing. Krousel‐Wood, Anderson, Fonseca, and Mauvais‐Jarvis provided critical comments on the study and reviewed and edited the manuscript. All authors have read and approved the final submission.

## FUNDING INFORMATION

This project was supported by a grant (National Institutes of Health K12HD043451) from the Eunice Kennedy Shriver of the Building Interdisciplinary Research Careers in Women's Health (BIRCWH) Scholar.

## DISCLOSURE

No prior presentation.

## Supporting information


**Figure S1.** Risk of cardiovascular disease associated with glycemic measures by race.
