# Supplementary material for: Sex differences in prognostic role of fasting glucose, Oral glucose tolerance, and HbA1c in diabetic cardiovascular disease
Source: J Diabetes. 2023 Feb 14;15(2):185–9. doi: 10.1111/1753-0407.13358 (PMC9934952; doi:10.1111/1753-0407.13358)
Supplement: Supplementary file 1 — Figure S1. Risk of cardiovascular disease associated with glycemic measures by race. [file JDB-15-185-s001.docx]

**Supplemental Figure 1 – Risk of cardiovascular disease associated with glycemic measures by race**

**Supplemental Fig 1.A. Prediabetes**

**Non-Hispanic Whites**

**Non-Hispanic Blacks**

**Supplemental Figure 1.B. Undiagnosed Diabetes**

**Non-Hispanic Whites**

**Non-Hispanic Black**

*<0.05; **<0.0001. Adjusted for age, race/ethnicity, education, BMI, smoking, blood pressure, cholesterols, menopausal status, uric acids, and anti-hypertensive or lipid-lowering medications.
